# Shared decision-making with older people on TReatment Escalation planning for Acute deterioration in the emergency Medical Setting - Observed (STREAMS-O): an ethnographic study

**DOI:** 10.1186/s12877-025-06893-7

**Published:** 2025-12-22

**Authors:** Bronwen E. Warner, Mary Wells, Cecilia Vindrola-Padros, Stephen J. Brett

**Affiliations:** 1 Division of Anaesthetics, Pain Management and Intensive Care, Department of Surgery and Cancer, Imperial College London, Exhibition Rd, South Kensington, London, SW7 2BX UK; 2 Department of Surgery and Cancer, Imperial College London, London, UK; 3 Department of Targeted Intervention, University College London (UCL), London, UK; 4 Department of Intensive Care Medicine, Imperial College Healthcare NHS Trust London, London, UK

**Keywords:** Shared decision-making, Treatment Escalation Planning, Cardiopulmonary resuscitation, Qualitative research, Ethnography, Focussed ethnography, Older people

## Abstract

**Background:**

Shared decision-making is increasingly expected in healthcare. Treatment escalation planning is a contingency for medical intervention in the event of acute deterioration in hospital. Treatment escalation planning guidelines in the United Kingdom reference shared decision-making but it is not clear to what extent this is feasible.

**Aim:**

To understand how shared decision-making takes place during conversations about treatment escalation planning between clinicians and patients in the acute medical setting for older patients with mental capacity to participate in decisions about treatment escalation plans.

**Methods:**

This was a focussed ethnography following a constructivist approach, conducted by a single researcher who is a medical doctor and qualitative researcher with input from a wider research team. Fieldwork took place April-July 2024 in an urban teaching hospital emergency department and acute medical unit. Data was collected as fieldnotes via participant observation, ad-hoc unstructured interviews, documentary analysis and case study analysis. Reflexive thematic analysis was conducted according to Braun and Clarke.

**Results:**

There were five themes, which, collectively, illustrated and explained challenges for shared decision-making with older patients in treatment escalation planning in the acute medical setting: ‘A cog in the hospital machine’, ‘One of many good decisions’, ‘A reluctant conversation’, ‘Gatekeeping the decision’, and ‘Unconstructive conversations’.

**Conclusion:**

Medical rather than shared decision-making was an inevitability in this context due to a complex set of interlinking factors, whilst patients assumed autonomy over their treatment escalation plan. This study questions the prevailing discourse that shared decision-making is always an optimal and achievable approach. Strategies to enhance shared decision-making in treatment escalation plans might include public education and encouraging clinicians and healthcare organisations to review treatment escalation plan decision-making practice. However, deliberation is required at policy level to determine expectations for different stakeholders’ authority over treatment escalation plans.

**Supplementary Information:**

The online version contains supplementary material available at 10.1186/s12877-025-06893-7.

## Introduction

Treatment Escalation Plans (TEP) are a contingency for future medical intervention, such as cardiopulmonary resuscitation status and admission to intensive care for organ support, if a patient loses the ability to contribute to healthcare decisions [1]. Population distribution is shifting towards older age [2] and the proportion of healthy life has reduced [3]. Factors associated with increasing age, such as frailty, co-morbidity and disability, influence health outcomes following acute illness [4, 5]. Attempts at life-saving treatment may be traumatic yet ultimately unsuccessful, or result in an unwished-for quality of life, and therefore, some patients may benefit from a less invasive approach. Older patients frequently experience acute hospital admission and many ultimately die in hospital [6]. Optimising the approach to planning for inpatient health deterioration with older people is therefore a pertinent topic.

Shared Decision-Making (SDM) is defined as a collaborative process where a patient and their healthcare professional make a joint decision about immediate or future care [7]. SDM is increasingly considered best practice in healthcare communication, including throughout the TEP process [7]. While various models for SDM have been proposed [8], the nature and ethical value of SDM are socially constructed and therefore fluid. Nonetheless, it is not clear whether SDM in the broadest sense is always feasible or desirable.

This research follows several decades of qualitative research interest in resuscitation acts and decisions. Much of the early exploration into treatment escalation practice and decision-making concluded in critique of the medicalisation of death and patient exclusion from decision-making [9–14]. Systematic review of primary studies exploring SDM in TEP for older patients has demonstrated that clinicians often tend towards medical, rather than shared, decision-making, but the reasons for this are not fully elucidated [15]. The attention in more recent research has been on seeking to improve patient involvement in TEP [16, 17]. While there have been recent ethnographies exploring TEP decision-making [18], we are not aware of any that have focussed on the SDM aspect with older patients.

This ethnographic study is part of a programme of qualitative research exploring the role of SDM in TEP for older people in the acute medical setting. This study aimed to understand: How does SDM take place during conversations about treatment escalation planning between clinicians and patients in the acute medical setting for older patients with mental capacity to engage in TEP?

## Methods

### Reflexivity

BEW conducted the fieldwork as part of a qualitative methods PhD and is a medical doctor with interests in internal medicine and geriatrics. MW is a professor of cancer nursing with experience in qualitative methods. CVP is a medical anthropologist. SJB is an ICU consultant with academic interest in clinical decision-making.

### Design

This is an ethnographic study. We chose this approach because of the complex and elusive nature of the social processes studied [19]. Given our ontological view of SDM (described in the introduction) as a social construct without fixed pre-social meaning, and because definitions of SDM continue to evolve [8], we considered decision-making between patients and clinicians in the broadest sense rather than evaluating against theoretical models. We take a constructivist epistemological stance, where we assume responsibility for how the ethnographer’s positionality and activities form a dynamic relationship with the field to generate and interpret ethnographic material [20]. We use the term ‘interlocutors’ to reflect the respectful and dialogic nature of the fieldwork [21]. This was a focused ethnography (FE) [22], a form of rapid ethnography [23]. Compared with traditional ethnography, FE is typically characterised as examining a more specific topic (a focused delineation of ‘the field’), being relatively time limited, relating to a specific community, generating more applied results and being conducted by insider researchers, but boundaries between traditional and rapid ethnographies are often blurred [24]. Typical of FE, there had been a long-term engagement with the field for several years through clinical and academic activities.

The study focused on activities in and around an emergency department (ED) and acute medical unit (AMU) in an acute NHS hospital in London, UK. Broadly, BEW sought to capture activities in different settings, at different times of day and with different interlocutors, in keeping with the ‘places, time, persons’ approach [25]. She went to clinical areas in the ED including ‘resus’, which is where the most unwell patients are looked after; clinical areas in the AMU; administrative staff areas in the ED and AMU; intermediate areas such as corridors and the hospital canteen. She spent time (8am-9pm) in the field to capture the overall pattern of the clinical day and when TEP conversations were mostly likely to be conducted by consultants. The researcher used purposive sampling to capture clinician perspectives from different healthcare professional (HCP) groups. Patients were aged ≥65 years, English-speaking and had capacity to be involved in TEP conversations and to consent to involvement in the study; NoK were included if patients wished. She sought diversity in patient demographics according to: clinical acuity, frailty [26], age, ethnicity (religion was also recorded if the participant agreed) and gender.

The fieldwork ended when there was ‘good enough’ material [27], guided by information power [28], data saturation (repeating ideas) [29], capturing a diversity of ‘times, places, persons’ and passing 100 hours in the field.

### Data collection

Fieldwork took place April-July 2024 and comprised the following:

A. Participant observation [30] and ‘hanging out’ (interacting with participants ‘in an uncontrived fashion as they go about their daily lives’) [31], to capture the culture and climate of healthcare decision-making, as follows: general ambiance, ward rounds, handovers, ad hoc conversations. BEW modified her approach depending on the context, along a spectrum from participating fully in the activities of those studied to being a ‘fly on the wall’ or non-participant observer [32].
B. Brief unstructured interviews (centred on experiences of TEP and whether and how SDM took place in TEP) with healthcare staff, including senior and resident doctors, staff and senior nurses, healthcare assistants, critical care outreach nurses, specialist nurses, occupational and physiotherapists, administrative team members and the patient safety team.
C. Documentary review of local guidelines, patient leaflets, staff educational resources and audit data on TEP and SDM.
D. Following clinical cases involving TEP on the post-take ward round. Usually this involved (1) observation: clinicians discussing the case; the consultation between patient and clinician; and the clinician discussion following the consultation, (2) brief interviews with the patient (+/-NoK – who were present if wished by the patient to support with the interview) and the clinician following the encounter regarding experiences with SDM and/or TEP, (3) reviewing the clinical record documentation.

Interview topic guides are included in Appendix 1.

BEW recorded her fieldnotes by hand whilst in the field, and transcribed and developed them electronically soon after. Field notes are denoted in the main text by being written in italics. Double quotation marks indicate verbatim (as closely as BEW could recall) quotations from participants. We have used ellipses in brackets to indicate where words have been removed; brackets with an asterisk indicate where detail has been added for clarity. Further detail on field note presentation is included in Appendix 2.

Patient age ranges and frailty (assessed by BEW according to the clinical frailty scale [26]) are included for cases in D.

### Ethical considerations

We designed the study in consultation with local clinician stakeholders and a patient and public involvement and engagement (PPIE) advisory panel representing diversity of ethnicity, sex and healthcare experience. Senior clinicians were gatekeepers to the field. Research Ethics Committee was obtained from the North East-York Research Ethics Committee 24/NE/0044.

Consent should ideally be a process which is dynamic and responsive to the needs of participants [33]. The overarching guiding principle was that consent should be based on ‘truthful and respectful’ exchanges between the researcher and participants/interlocutors [34]. Participant information sheets were available to all. BEW obtained written or verbal consent depending on the nature of involvement. We discussed this with the PPIE and clinician stakeholder groups, who felt that introducing a bureaucratic process would be unnecessarily disruptive during some clinical activities. Consent for the various aspects of the study (A-D in Data Collection, above) was as follows: patients and clinicians in (D) gave written consent; clinicians in (B) gave verbal consent; senior clinician gatekeepers gave consent in (A). The researcher’s role was transparent and no personally-identifiable details were recorded.

### Analysis

We conducted reflexive Thematic Analysis according to Braun and Clarke [35]. BEW familiarised herself with the data iteratively along with her analytic memos. She coded the fieldnotes and documents inductively using semantic and latent codes. She clustered codes with patterned meaning using mind-mapping and generated initial themes. Candidate themes together with field note examples were discussed iteratively amongst the wider research team. At this stage, BEW revisited the data materials again in full and began writing and drawing on direct fieldnote extracts to test how ideas within each candidate theme fitted together. The themes were thus developed and revised so that each one had a central organising concept. In the refining, defining and naming stage, we sought to tell a unifying narrative and to map back to the shared decision-making focus of the research.

## Results

BEW conducted approximately 107 h of fieldwork.

First, we describe the observed hospital context in which TEP decisions are made. The findings are then presented as five themes, which illustrate and explain challenges to SDM in TEP: ‘A cog in the hospital machine’, ‘One of many good decisions’, ‘A reluctant conversation’, ‘Gatekeeping the decision’, and ‘Unconstructive conversations’. Theme names and definitions are presented in Table 1. We use an integrative strategy, where excerpts and interpretation are woven together [36]. Extended and additional extracts are included in the Appendices 3&4.

**Table 1:** Theme names and definitions

THEME	THEME DEFINITION
A cog in the hospital machine	*TEPs are viewed by hospital staff as an indispensable cog in the hospital machine which benefits patients and the wider clinical team. TEP decisions are used to triage management of deteriorating patients and protect from excessive treatment. To be useful*,* TEPs need to be complete*,* unambiguous and visible.*
One of many good decisions	*Healthcare decisions are complicated*,* nuanced and instinctive. Doctors make numerous decisions for (rather than with) patients. They aspire to high standards of care*,* mindful of a sense of personal responsibility and external scrutiny. TEPs are considered a continuation of this decision-making approach.*
Gatekeeping the decision	*Clinicians are gatekeepers to the TEP conversation. There are various systemic and local drivers for the TEP to be completed*,* but clinicians use their discretion about whether*,* when and to what extent TEPs are made and discussed.*
A reluctant conversation	*TEP are seen as a difficult topic and potential source of conflict. Patients can find the conversation distressing. Clinicians embark upon the conversation reluctantly*,* seeking to negotiate towards an efficient and amicable conclusion in alignment with the medical view.*
Unconstructive conversations	*Decision-making authority is viewed variously by different members of the clinical team*,* patients and NoK*,* and there is lack of consensus on who holds this authority. Some patients have pre-existing ideas about CPR*,* but most do not appear prepared for a nuanced*,* detailed conversation. These factors do not predispose the conversations between patients and clinicians towards shared decision-making where the conversation is meaningful for the decision. The conversations do not necessarily improve patient or clinician understanding of the other’s views and decisions do not always reflect patients’ preferences.*

### Context: a pressurised environment

Whilst conducting the fieldwork, it was clear that the human and physical environment influenced many aspects of TEP and how SDM could occur. The hospital was pressured with workload, staffing, bed availability, patient acuity, organisational processes and clinical complexity. Multi-tasking was demanding for all staff groups. Police were a frequent sight in the ED, and while they usually accompanied acutely confused and physically aggressive patients, their presence hinted at the conflict which frequently seemed to be just around the corner in this often fraught and high stakes setting. TEP conversations took place amidst this backdrop, sometimes challenged by space, noise, interruptions and distractions.

### Themes

#### Theme 1: A cog in the hospital machine

##### Definition

*TEPs are viewed by hospital staff as an indispensable cog in the hospital machine which benefits patients and the wider clinical team. TEP decisions are used to triage management of deteriorating patients and protect from excessive treatment. To be useful*,* TEPs need to be complete*,* unambiguous and visible.*

The TEP was a priority piece of information in the clinical day. In clinical handovers, it was often the third key detail: *“This is a 72 year old male for full escalation”.*

Clinicians appeared sympathetic to older patients’ vulnerability in hospital and believed that TEPs improved care. Doctors felt that it was wrong to treat patients too aggressively if they seemed unlikely to benefit. Nurses said that once there was clarity about a TEP they could focus on comfort. The benefits of TEP were described in the Trust’s mandatory on-line educational material, in terms of preventing futile attempts at resuscitation, helping patients and NoK to understand and prepare, ensuring appropriate resource use, encouraging decisions before a crisis and allowing a peaceful death.

If a patient who is ‘for CPR’ arrests, cardiac arrest teams are alerted and assembled instantaneously, from staff members with diverse responsibilities throughout the hospital. At the beginning of each shift, the ‘cardiac arrest huddle’ met to assign team roles. As they dispersed, there was an oft-used farewell, *“well*,* let’s hope we don’t see you”.* This seemed to hint both at sadness inherent in loss of life but also the disruption a cardiac arrest call causes in an already busy shift, redirecting staff from admitting patients, urgent reviews, the operating theatre and breaks.

TEP also triaged the next steps in a deteriorating patient’s management. *In the AMU office*,* one of the SHOs flagged to the consultant a new patient from ED. She said she wanted to mention him because “he’s just come round*,* just because he’s for full escalation*,* you know*,* and he’s sick.”* There was a sense that higher intensity treatment might be possible. By contrast, in the morning AMU handover the medical and nursing teams were discussing a patient who had been unwell overnight, but was already receiving maximum treatment according to her TEP: *The consultant recapped the likely diagnosis*,* and checked*,* “what is her escalation?” Hearing that she was ward-based*,* she continued*,* “ok*,* so we are doing furosemide*,* antibiotics*,* chest physio*,* saline nebs”.*

Not *“wrapping up”*, as one consultant termed it, a TEP on admission was perceived by doctors not only to harm patients but to expose colleagues. Several resident doctors emotionally described scenarios where they had struggled with a deteriorating patient whose TEP was not clear. Handing over an unwell patient without a TEP was sometimes viewed critically.

It seemed important that the TEP was clearly communicated, but this was not always achieved. Nurses were particularly concerned about this, anticipating that they might be the first person to find an unresponsive patient. The nursing handover sheet had a list of patients it had been decided were not for CPR but in the high-turnover ward they did not consider this reliable. A code appeared on the electronic patient record (EPR) if a patient had a previous TEP decision made in the hospital: nurses understood that they must commence CPR if these patients arrested, even if the previous decision had been DNACPR; resident doctors sometimes assumed that the label equated to DNACPR and deprioritised completing a new form. TEP information also did not appear to flow easily between hospital and primary care; such fragmentation created opportunity for confusion.

#### Theme 2: one of many good decisions

##### Definition

*Healthcare decisions are complicated*,* nuanced and instinctive. Doctors make numerous decisions for (rather than with) patients. They aspire to high standards of care*,* mindful of a sense of personal responsibility and external scrutiny. TEPs are considered a continuation of this decision-making approach.*

Across professional groups, people seemed to take seriously their responsibilities to deliver a high standard of care and wanted the best for patients. *On the wall outside the AMU*,* there was a colourful infographic entitled ‘Pathway to Excellence’*^®^. *They had used a London underground metaphor*,* and it was enthusiastic*,* positive*,* motivational.* Staying late and a soldierly stoicism when describing challenges seemed part of the local culture.

There was a sense of scrutiny over clinicians’ clinical decision-making practice and professional behaviour from peers, seniors and ultimately legal teams. Staff strived to perform well and conform in their working world. They appeared conscious of making defensible decisions and protecting themselves through mechanisms such as rigorous documentation in case of future investigation. In the morning AMU handover, *the night registrar mentioned that there had been one sick one but he’s now in ICU. The consultant (…) said it is Trust policy to call the consultant about anyone going to ICU*,* otherwise if there is a complaint and they don’t know about the case it is not good. (…) She was stern*,* unrelenting.*

Clinicians seemed eager that their practice be in line with guidance and were keen to have workable rules to follow. They appeared aware that TEP was potentially controversial and subject to future legal enquiry; perhaps in response, senior staff sometimes used fluent, pre-rehearsed phrases when describing TEP. In general, doctors frequently quoted that patients without a TEP are by default for full escalation, that it was imperative to discuss with patients before formally documenting a DNACPR, and that CPR is a medical decision. There appeared less consensus on other guidance, such as the process to follow for patients with a DNACPR decision made in primary care. One *asked me the ‘rule’ about community DNAR(…) She seemed controlled*,* calm*,* keen to learn the ‘correct’ way of doing things. We’d talked about the evidence around ulcers with steroids earlier and her tone had been similar.*

In this context, the hospital day was filled with decisions, few of which appeared straightforward. A web of interacting considerations, many of which were imprecise, was untangled and interpreted to produce an output which was by necessity clear cut.

The PTWR was a thorough process which clinicians seemed to take seriously and wanted to do well. Away from the patient’s bedside, resident doctors presented the case to the consultant and they reviewed the notes in detail. The aim appeared to be deciphering the clinical complexity, often inherent in cases of older, multi-morbid patients, to form a coherent clinical picture and determine management. The beginnings of a many-stage plan were drafted before the medical team reviewed the patient. There seemed to be a gut feeling, assimilated through experience, which was the glue holding clinical facts together. Clinicians used their clinical experience to interpret the nuance of clinical details and assessments were often qualitative as well as quantitative, such as in this case where chronological vs. physiological age were discussed. *“She doesn’t look 81”*,* the clerking resident doctor remarked to the take consultant. “No*,* she doesn’t*,*” the consultant agreed. “Although the face*,* you can see*,* alcohol”. She actually looks quite “robust”*,* they agreed*,* the word spoken ‘robustly’ and the consultant miming arm strength with a skier holding poles pose.*

This complex, instinctive, iterative decision-making process was similarly at play for treatment escalation plans. Clinicians considered acute and chronic physiology, functional baseline and age, and synthesised these together implicitly to determine a TEP. They appeared intent on making what they deemed ‘good’ decisions. *Discussing resus status on the PTWR*,* the night registrar said*,* “He seems robust but elderly”. The consultant remarked*,* “he doesn’t have a package of care*,* he had all the prostate surgeries. We could keep him for full escalation for now unless the collateral reveals something else”. “He said he was into sports but I’m not sure I believed him*,*” the registrar added. They made a plan to talk to his wife about his baseline function and cognition then decide escalation status accordingly. Later on*,* after talking with the patient*,* the consultant remarked*,* “it’s probably a stress fracture. To be honest*,* if he walks that much*,* I’d probably keep him for full escalation”.*

There appeared a general clinical consensus about what the TEP should be for most patients; this approach was also used to sense-check others’ decisions. The medically acceptable TEP seemed to derive predominantly from a combination of clinical data and intuition, and it was hard to pinpoint precisely what determined the eventual outcome. Doctors often deferred to those with greater experience. Resident doctors sought to learn the status quo of what TEP decision was likely to be made: efficiently documenting the post take ward round, they sometimes drafted *‘DNAR – ward based’* in anticipation of the decision. They also formulated TEP decisions in line with what they had seen colleagues decide and embarked independently on conversations with patients. That said, several commented that some seniors had different approaches to TEP thresholds and they found this confusing.

Clinicians appeared to consider TEP their responsibility. I asked a resident doctor, referring to a conversation she described with a patient, *Why do you say it’s a medical decision? She responded simply*,* “So they don’t feel responsible for their life and death”.* Several clinicians explained that it was important not to frame TEP as a question to spare patients or NoK the burden of the decision, especially if the older patient was anticipated to be more vulnerable to distress. There were instances where the ‘right’ TEP was uncertain and clinicians sought colleagues’ opinion, but involving patients in this uncertainty was rarely considered. *One resident doctor mentioned to me an ‘interesting case’ where a lady in her 70 s has reversible pathology but was very frail. There had been lots of discussions*,* but in the end they decided ‘full escalation’. Discussions with the patient*,* I asked? Oh no*,* they were outside*,* not discussed with her.* The DNACPR decision was mostly one to be communicated *to* the patient, kindly but firmly breaking the bad news. As one consultant told me, passionately, *it should be a medical decision*,* we need “more paternalism”. “You wouldn’t be able to tell a car mechanic to resurrect an old car!” or demand chemo or dialysis*,* she argued*,* so why is CPR any different?*

Ultimately, clinicians remarked that the decision about how invasively to treat would be made by the team responsible for intervening in the event of deterioration. This might be the on-call internal medicine team at the initial response, or the intensive care team who would decide whether to accept the patient to the ICU. TEP decisions were about how much treatment might be *considered*, rather than *delivered*. Therefore, no matter the patient’s view, clinicians inevitably held authority for what would happen if they became more unwell.

#### Theme 3: gatekeeping the conversation

##### Definition

*Clinicians are gatekeepers to the TEP conversation. There are various systemic and local drivers for the TEP to be completed*,* but clinicians use their discretion about whether*,* when and to what extent TEPs are made and discussed.*

There was expectation at an organisational level and outlined in policy documents, which had increased following the covid pandemic, that TEP decisions be made and documented early in the admission for patients where *‘cardiac or respiratory arrest (is) a possibility’*. Decisions usually took place on the PTWR, whose documentation included an acronym to prompt completion of key tasks including the resuscitation status. Local audits measured rates and timing of TEP completion.

However, clinicians used their discretion on when to make TEP decisions. The conversations were too time-consuming or potentially rife with distress and conflict for most clinicians to be willing to embark on one with every patient. Some consultants appeared to discuss routinely and some infrequently. Sometimes, consultants delayed making the TEP, awaiting clinical information or out of sympathy for the patient. While many decisions were made, staff from different groups often mentioned cases where they felt TEPs had been wanting.

Clinicians appeared more likely to discuss TEP and complete a detailed plan with patients they anticipated would fare poorly following invasive treatment due to their baseline condition of age, frailty and co-morbidities, especially if the patient more acutely unwell. Local audit data showed that most TEP decisions formalised on the EPR were *not for* rather than *for CPR*. The patient information leaflet on CPR and TEP appeared directed at the DNACPR discussion, emphasising that *‘the success rate is low’*. For patients where clinicians considered ‘full escalation’ appropriate, TEP was usually not discussed.

Patients actively deteriorating, or those anticipated to become unwell imminently, were the highest priority for TEPs. Nursing staff advocated for patients to ensure that they were receiving treatment, and, on a practical level, one ED nurse remarked that she needed to know whether to go and get the ‘crash’ trolley which contains the equipment needed to manage a cardiac arrest.

Resident doctors sometimes prompted consultants to make TEP decisions. They seemed trained to believe that early TEPs were inherent to good practice. *The SHO*,* mildly forceful*,* fingers poised to type*,* asked*, “so what do you want for (the TEP*)?” The consultant replied,* quickly*,* “DNAR.” Then*,* kindly*,* offhand*,* “not for ICU. They won’t take an 85 year old. Consider NIV”* (non-invasive ventilation, a form of respiratory support*). *She moved off*,* the ward round over*,* towards the AMU office where the daily board round would be starting. I stayed with the SHO who seemed a bit put out. “It’s not ideal*,* is it?” she said. “We can’t talk to her*,* don’t know her baseline but she doesn’t have carers… I tried to get a decision. The consultant just said – ” “They won’t take an 85 year old” I filled in. She laughed hollowly*,* “Yeah exactly. We’re ageist. What if she deteriorates? It’s just kicking the can down the road. I’ve been trying*,* we had one earlier who’s been here a few days*,* bedbound*,* definitely should be DNAR*,* but she just said*,* ‘let’s see what happens’”.*

Clinicians also selected how much TEP detail to explore with patients. Often, they only discussed CPR, rather than a full set of escalation options, sometimes alongside reassurance about management that would continue. For other treatments, such as organ support, there was variable discussion with patients or documentation of specific details in the notes.

#### Theme 4: A reluctant conversation

##### Definition

*TEP are seen as a difficult topic and potential source of conflict. Patients can find the conversation distressing. Clinicians embark upon the conversation reluctantly*,* seeking to negotiate towards an efficient and amicable conclusion in alignment with the medical view.*

The TEP conversation was generally viewed as challenging. A handful of staff spoke with enthusiasm and passion about how important TEPs are for patient care and shared their tips for how to approach the conversation. Most staff appeared to consider TEP a difficult duty. *One registrar remarked*,* “I try to avoid them (…) I just don’t like to tell nice people sad things”.* TEPs had many negative connotations for clinicians: potential for a ‘bad death’ if the decision was not secured; scrutiny from the wider team; a daily brush with the unwelcome reality that medicine does not always help; and negative reactions from patients and NoK. They often talked about TEPs and conflict in the same breath and recalled difficult conversations from years previously. Sometimes, clinicians anticipated conflict prior to commencing the TEP conversation, due to the tone of the preceding interaction or because of predicted cultural differences. There were text and pictorial patient information leaflets to help navigate conversations about TEP and CPR, although I never saw these handed out. One stated, seeming to hint at the potentially taxing conversation, *‘Your views are important and we want to involve you in decisions about your treatment. We recognise that these discussions can be distressing but your doctor will raise these issues as sensitively as possible.’*

Treatment escalation plans herald a possible deterioration and death; they are, by definition, serious and potentially sad. As one patient (85-90yrs, severe frailty) said after a TEP conversation, *“I did feel a bit ‘urgh! Am I dying?!’ I try not to think about these things. You always hope it’s something that happens to someone else*,* but now*,*” gesturing to his surroundings*,* “it’s me!”* Staff identified many potential sources of upset from TEP conversations. Patients and NoK might not understand treatments and their implications. A TEP conversation could be interpreted as hinting that death was imminent, such that TEPs took away hope. Some patients or NoK believed that restrictions were made despite available treatment, meaning that they felt clinicians were giving up on them. According to a conversation with a senior AMU nurse, *patients who need to go to ICU are too sick*,* they’re not able to have that conversation*,* but she’d never had anyone who said they didn’t want to go to ICU. For the ones where it’s more in advance*,* “patients come into hospital*,* they want to get better*,* sometimes in time they do*,* sometimes they don’t. But some of them feel if you talk to them about it*,* you are taking away hope”. She also said that she was “very aware” that some people think having a not for CPR form (she pointed at the EPR header) documented means – she mimed brushing away with the back of her hand – they don’t get any treatment.*

Clinicians therefore sought to manoeuvre the conversation to an agreeable conclusion without dwelling too much on the distressing or contentious topic. There appeared to be a stereotyped approach. TEP conversations almost always came at the end of the PTWR. Clinicians explained that this helped to ensure some rapport, but sometimes the topic appeared disconnected from the earlier discussion. Embarking on the conversation, clinicians often adopted a new physical position such as kneeling or leaning in. They delivered a warning shot, denoting a change from the conversation about the immediate health problem to the TEP conversation. Many clinicians seemed to have developed a practised series of phrases, which they used for most conversations with some adjustments for the individual case. There was sometimes a sense that clinicians felt the need to deliver certain information regardless of whether the patient was able to take it on board. Once the TEP was secured, patient agreement apparently in place, many clinicians quickly ended the conversation. A consultant *explained that because of the infection in the blood stream the patient could become more unwell and then we would not resuscitate. The patient* (85-90yrs, moderate frailty) *heard him out then said*,* with a twinkle in his eye*,* “that is what I would have said”. The consultant turned round to the scribing junior. “I think that’s straightforward”. Almost immediately*,* he got up*, conversation ended. I sensed some relief (from the clinical team*).

The discussions often had a persuasive tone, aiming to bring the patient to a point of agreement with the medical perspective that treatment limitation was appropriate. *I asked a consultant whether*,* in uncertain cases*,* she ever asks the patient. It seemed like a non-question. “About escalation? No! I tell them. You know*,* it’s a physiological decision - if they have tight aortic stenosis. it’s quite complex”. She would discuss CPR*,* she said*,* and described telling them her decision. The ED consultant joined in. “I ask them*,* ‘if you’re DEAD do you want us to try to bring you back with (miming) chest compressions and broken bones?’” The AMU consultant agreed*,* “yes*,* they probably agree*,* she would probably agree.”* It appeared difficult to embark on a conversation without a medically-proposed TEP at which to aim.

Patients and clinicians both agreed that rapport was important for trust and to reduce conflict (even if it did not guarantee agreement). Patients talked about ‘care’, being ‘put at ease’, ‘warmth’, the ‘tone’. One said she aborted the TEP conversation because she did not feel rapport with the consultant. Several nurses remarked that they had witnessed difficult conversations where they perceived the medical communication to have been too “*harsh*”.

Nurses’ role in TEP conversations was usually supportive. They volunteered hospital mandatory training as a source of information about CPR decisions. Nurses, therapists and other members of the multidisciplinary team might have ideas about what the TEP was likely to be, but the difficult decision and conversation were considered the remit of doctors, and nurses comforted patients or recapped explanations afterwards. *A staff nurse told me that sometimes patients or family are unhappy after a DNACPR discussion (…) When they tell him this*,* he says*,* “the doctors have studied your case to make the decision. They do this because it is the best decision*,* otherwise it would not be good for you*,” then distracts them with the offer of refreshment. They are usually calmer after that. I joked that this is very helpful for the doctors. (He said*),* “Yes*,* when the doctor comes back it is better for them*,* we are all in it together.”*

#### Theme 5: unconstructive conversations

##### Definition

*Decision-making authority is viewed variously by different members of the clinical team*,* patients and NoK*,* and there is lack of consensus on who holds this authority. Some patients have pre-existing ideas about CPR*,* but most do not appear prepared for a nuanced*,* detailed conversation. These factors do not predispose the conversations between patients and clinicians towards shared decision-making where the conversation is meaningful for the decision. The conversations do not necessarily improve patient or clinician understanding of the other’s views and decisions do not always reflect patients’ preferences.*

Clinicians had diverse views on whether patients or clinicians had authority for TEP decisions. An ED nurse remarked, *“We are a democratic– well*,* I think we are (he laughed) – 21 st century country. You know if it were me*,* I would want to make the decision. Otherwise*,* how is it ethical?”* By contrast, *the ED consultant said firmly*,* in a slightly sing song tone as if she had repeated this many times*,* “whether he’s for CPR or not is a MEDICAL DECISION”.* Some people suggested, “both?” but on closer questioning described a scenario where doctors explained so that patients agreed. Patient information leaflets said that CPR was a medical decision but that patients should be involved.

Patients generally assumed that they would have the final say on TEPs. Many had some pre-conceptions of CPR and an opinion about whether they wanted it or not. That said, on closer questioning, few were able to volunteer potential consequences of treatment. In general, even if patients had views on TEP, most did not appear to anticipate the conversation when it was broached and were not prepared for an in-depth discussion. In one example, there was a female patient (85 + yrs, mild frailty) with pneumonia who, together with her family, was adamantly in favour of CPR. The initial decision suggested by the medical team for a DNACPR was firmly rebuffed and subsequently the conversation was revisited time and again over the weekend as she deteriorated. Speaking with the patient and her family after a hostile initial interaction between them and the medical team, *They had experience of CPR conversations because the patient’s husband*,* brother and daughter had been DNAR*,* so the concept was familiar*,* she’d made the decision for her husband. (…) She said she is interested in medical things*,* “although I’m not a doctor!”*,* like magazines and the television. They make CPR look ok. She doesn’t think her friends would have thought about it*,* but it doesn’t upset her. I asked her what she knew about what happens during CPR and its aftermath*,* she said*,* “not much really.”*

Clinicians followed an implicit algorithm whereby they discussed TEP with patients if they were deemed to have mental capacity and otherwise with NoK. However, capacity was described as a ‘grey’ area. Capacity was not often formally assessed prior to TEP, and it sometimes appeared that patients who had been involved in the conversation did not have capacity. Some clinicians also felt that most patients were unlikely to grasp fully the complexities of TEP decision-making even if they formally had capacity, meaning that clinicians were better placed to make decisions. The concept of ‘best interests’ decisions (technically reserved for patients without capacity) seemed, for many clinicians, to be applicable to all patients when making TEPs. However, patients and NoK did not necessarily have the same appreciation of what capacity is; in contrast to the clinicians’ algorithm about capacity, many assumed that patients would be involved in decisions regardless. Conflict with NoK relating to the relative authority of older patients, clinician and NoK over TEP decisions was frequently referenced by clinicians.

Clinicians sometimes used their experience to interpret patient or NoK demeanour to inform whether or how to discuss or decide TEP. *A patient’s* (90 + yrs, severe frailty) *daughter asked to speak with the consultant about the DNACPR decision. She said that she wants us to keep him alive if it’s during the day so she can get there*,* she just wants him to die in his sleep at night. I was silently uncomfortable; this didn’t seem logical. The consultant nodded*,* encouraging*,* ignored the part about keeping him alive*,* “that’s right*,* we’ll treat what will work but what I don’t want to do is things which will cause pain and distress*,* but which won’t work”. Everyone nodded*,* agreeing.*

Observing TEP conversations and then speaking to patients afterwards, it became apparent that the clinician and patient interpretation of the discussion (and consequently the documented TEP plan) frequently did not match. Sometimes this occurred for patients whom clinicians made DNACPR: a clinician believed that they had communicated the medical decision for DNACPR but the patient (75-80yrs, moderate frailty) thought that the decision was still theirs to finalise and did not plan to agree; or a patient (85-90yrs, severe frailty) interpreted the DNACPR conversation as meaning that the treatment would not be necessary, rather than that it would not be attempted. In these cases, DNACPR may have been consistent with the patient’s goals of care, but not with their stated TEP preference. Mismatch between the clinician’s impression and patient’s wishes also occurred with patients made for ‘full escalation’: a patient (85-90yrs, mild frailty) resisted the conversation as inappropriate for her current admission and due to lack of rapport, and the decision defaulted to full escalation, but she later said she did not wish to be resuscitated due to concerns about dependence; a patient (70-75yrs, mild frailty) said she wished to have CPR but later explained that she did not want to be ‘revived’ as she felt unwell and ready for death; a patient (75-80yrs, mild frailty) agreed to the consultant’s assumption that she would want her heart restarted, but later appeared concerned about the possible consequences. For these cases, the clinician was willing to offer CPR and other invasive treatments but did not explain what these involved and their potential aftermath, meaning that patients’ default response of wanting CPR might conceivably have changed with more discussion.

## Discussion

This ethnographic study aimed to understand how shared decision-making takes place in treatment escalation planning in the acute medical setting. As far as we are aware, this is the first study specifically to explore this research question using ethnographic methods. We demonstrate that medical, rather than shared, decision-making currently predominates and may even be an inevitability due to a complex set of interlinking factors.

There is an expectation in the UK that health decisions should be shared between patients and clinicians, and the Resuscitation Council UK and Intensive Care Society reference SDM in TEP standards [17, 37]. What constitutes ‘sharing’ is ill-defined. First, there is the issue of who should hold ultimate authority in cases of disagreement. Legal rulings mandate that patients are at least informed of CPR decisions [38, 39]. At present, clinicians are not required to provide treatment they consider inappropriate but there is broader move in case law towards increasing patient autonomy [40]. This study demonstrated a fundamental difference of opinion between patients and clinicians about where authority should lie. Beyond this, SDM conversations should ideally seek a synergy of stakeholders’ knowledge and opinion to produce an enhanced decision. The study did not find that this goal is currently realised. The reasoning underlying these findings is complex, as outlined in the following discussion.

In ‘A cog in the hospital machine’, an unambiguous TEP was integral to hospital functioning. Used so frequently, in time-limited exchanges, the ‘resus status’ assumed a somewhat paradoxical role as a label which was both high-priority and life-defining but also generic, abbreviated and unemotional. There was little space for its implications on life or death, or nuances like patient priorities or subtle variations in exactly how much treatment might be attempted. This has parallels with Cohn’s observation of CPR as a ‘master status’ [41]. Indeed, previous ethnographies have critiqued the dominance of the biomedical narrative in life and death decisions [10, 13]. More recent studies have also remarked how the pressurised environment can directly impact on the quality of TEP conversations [42]. This was echoed in the broader decision-making climate observed in this study, but timely TEPs also appeared to have advantages, providing structure in a fast-paced and unpredictable environment and being believed by clinicians to improve patient care. Nonetheless, taken together, this theme illustrates that there was little capacity for TEPs to misalign with clinician or organisational expectations or to take too long to make, both of which challenge sharing the decision.

In ‘One of many good decisions’ and ‘Gatekeeping the conversation’, clinicians assumed authority for the TEP. They considered both the plan anticipating deterioration and medical response in the event of deterioration to be the remit of clinicians; they triaged whether and in what detail to make a TEP according to clinical factors and their personal clinical approach. As with other studies [17, 43, 44], heuristics were an integral part of decision-making. Clinicians’ commitment to medical decision-making is a pervasive idea in the literature [14, 45–48], including in TEP research [9–14]. This appears to derive from good intentions to protect vulnerable patients and to take personal and professional responsibility for complex decisions. This study highlighted some challenges to shifting the narrative from a medical to sharing one. First, clinicians were committed to making a ‘good’ TEP, but a shared decision might produce a different output, making clinicians reluctant to relinquish authority. Next, TEP decisions took place as part of a complex, fast-paced, intuitive process amidst a web of other decisions, such that sharing the decision with patients could disrupt this flow and feel unworkable. Finally, clinicians felt that it was inappropriate or even harmful to discuss TEP with some patients, but this meant that sharing the decision was foreclosed.

‘A reluctant conversation’ and ‘Unconstructive conversations’ explore the challenging nature of TEP conversations but their limited impact on the decision itself. Framing the conversation, it was remarkable that patients and clinicians often held fundamentally opposing views about who had authority over the TEP: patients believed that they should make the decision; clinicians largely assumed medical authority. The goal of healthcare decision-making communication generally is debated, with some prioritising shared understanding [49] and others maintaining that this differs from SDM [50]; existing UK TEP guidance does not always delineate between the two. As with Timmermans [9], patients appeared to have a general disposition towards maximal escalation, but like other studies [16, 51] lacked specific knowledge about TEPs and did not anticipate the conversation when meant that they were ill-prepared for a meaningful exchange. In addition, patients usually lacked knowledge of what maximal escalation and its consequences actually involved. The conversations themselves, as in previous accounts [14, 45, 46, 52, 53], were persuasive, and clinicians sought to negotiate the conversation to a hopefully rapid and amicable conclusion in line with the medical view. Conflict was also prominent; this is consistent with other studies which have attributed conflict to clinicians’ reticence to deliver harmful, non-beneficial intervention juxtaposed with patient concerns that clinicians are withholding treatment, and TEP’s essentially emotional nature [14, 43, 45–47, 52–58]. Overall, the conversation did not appear destined to be a shared decision, and in fact shared understanding was also often not achieved.

### Implications

This study describes a clinical decision-making climate at odds with societal and academic expectations for SDM. Through in-depth exploration, we present insights into how TEP conversations take place in the acute medical setting, juxtapose different stakeholders’ views on sharing the TEP decision, and present a critique of why SDM is constrained.

In terms of analytical implications, this study questions the prevailing academic discourse which suggests that SDM is inarguably the best healthcare communication approach. We demonstrate that there are systemic, interpersonal and decision-related factors which might preclude SDM in some circumstances. While some of these factors might be addressed to promote SDM, others will be either immutable or the net benefit of insisting on SDM may not advance the greater good.

From a patient perspective, greater public understanding about TEP would enhance patients’ preparedness to engage in meaningful conversations and might empower patients who do not wish to be resuscitated to volunteer this information even if the clinician does not broach the topic. This could be approached through increased dialogue in the media, public health messaging and conversations with community-based clinicians. These channels would need to engage older people, for whom TEP decision-making is especially pertinent, and also NoK, who often play an important part in supporting with TEP conversations.

From a healthcare perspective, a more open approach to what a ‘good’ TEP looks like and to timing the conversations might facilitate a more shared approach. This would require intervention across multiple domains including in clinical training, local and national guidelines, and performance-management reporting. There may remain circumstances in which clinicians do not consider SDM to be possible. Currently, there appears to be a categorical approach for most cases whereby patients are either assumed to be for full escalation or informed that treatment limitation would be more appropriate. Promoting recognition of selected, more ambiguous cases where SDM could be most useful, in accordance with existing guidelines [37], may be beneficial, but we note wider environmental constraints.

Finally, we reflect on the diversity of views on TEP authority expressed in this study to remark on the need for iterative policy and legal deliberation on where TEP decision-making authority should lie. In the context of evolving social norms trending towards increased expectations for patient autonomy, healthcare developments which increase the potential for what medical intervention can offer, and the assisted dying conversation, we suggest that policy makers should anticipate active debate on TEP decision-making and be prepared to deliberate accordingly on SDM feasibility and desirability.

### Limitations

This study was conducted at one centre, to facilitate relationship-building and in-depth exploration of the field. Local cultures are likely to differ between hospitals, meaning that readers should interpret the findings in the context of their own setting. Nonetheless, national laws and guidance on TEPs are UK-wide, meaning that there will likely be some transferability to other acute settings. Many of the contributing physicians had worked at numerous other hospitals and this will have influenced their attitudes and approaches, meaning that, although there may be local nuance, normalisation is likely to have occurred across a wider institutional base.

The study focussed on conversations made in anticipation of possible future deterioration. Conversations were mostly observed with stable patients, albeit with a range of pathologies from relatively minor to life-threatening. Nonetheless, TEP conversations do sometimes take place whilst patients are actively deteriorating, and these were not captured. Fieldwork was conducted during the day, to study decisions made by consultants with ultimate responsibility for the TEP decision. This means that any differences relating to nighttime conversations have not been captured. However, the acute and emergency medical settings operate a continuous service and staff work shifts across all time periods, meaning that the interlocuters who shared their views likely drew on perspectives from other aspects of their experience.

We intended to study TEP decisions and conversations with patients with diverse ethnicities and religious beliefs. While, in keeping with the diverse population served by the hospital, there were episodes relating to patients from different backgrounds, fewer patient interviews transpired with patients from non-White backgrounds than expected. There did not appear to be a pattern explaining this. Future work would ideally achieve a more diverse range of patient interlocuters. Clinician interlocuters did seem to reflect the multicultural staff population.

We did not focus on patients formally lacking capacity to make TEP decisions, as this was not within the remit of the study. However, capacity considerations often appeared important and so some aspects of TEP decision-making for patients without capacity have been discussed. Future work would be important to explore this more specifically.

We considered TEP decision-making between patients and clinicians pragmatically and in the broadest sense, rather than based on SDM communication frameworks, reflecting our view of SDM as a social construct with numerous defining models.

## Conclusion

This ethnographic study aimed to understand how treatment escalation planning conversations take place with older patients in the acute medical setting and how context influences shared decision-making. Shared decision-making did not appear to be achievable, due to a constellation of decision-related, individual, interpersonal and contextual factors. Clinical TEP decision-making was focused on achieving best care for older patients, but through ‘good’ rather than ‘shared’ decisions as defined by clinicians and the hospital context. These findings are at odds with the geriatric medicine narrative and societal moves towards patient empowerment in healthcare. We present a critique of SDM in TEP alongside a discussion of how SDM could be enhanced. These findings have relevance to older patients, clinicians, academics and policymakers.

## Supplementary Information

Supplementary material 1.

## Data Availability

The datasets generated and/or analysed during the current study are not publicly available due to their potentially sensitive nature but may be available from the corresponding author on reasonable request.

## Glossary

Acute Medical Unit (AMU): a hospital ward where patients admitted under the medical team are typically treated for up to the first 72 hours of their hospital stay
Cardiac arrest: sudden cessation of heart functioning
Cardiopulmonary resuscitation (CPR): an intervention following a cardiac or respiratory arrest which attempts to restart the breathing or restore the circulation. It can involve chest compressions, delivery of electric shocks from a defibrillator, injection of drugs, and ventilation of the lungs
Do Not Attempt Cardiopulmonary Resuscitation (DNACPR): A decision not to attempt CPR in the event of a cardiac arrest
Emergency Department (ED): the area of the hospital where acutely unwell patients are first assessed and managed
Electronic Patient Record (EPR): the digital platform on which clinical information is documented
Full escalation (FE): a term used to describe a TEP where the patient would be admitted to the ICU and receive CPR if required. Conversely, ‘ward-based’ care, where a deteriorating patient would be cared for on a normal hospital ward and not admitted to the ICU or receive CPR
Next of Kin (NoK): term for persons nominated by the patient to represent them
Post Take Ward Round (PTWR): review by a medical consultant of patients admitted via the medical take. The post take consultant holds ultimate professional responsibility for these patients
Resus(citation) status: whether a patient would receive CPR in the event of cardiac arrest
