# Supplementary material for: Shared decision-making with older people on TReatment Escalation planning for Acute deterioration in the emergency Medical Setting - Observed (STREAMS-O): an ethnographic study
Source: BMC Geriatr. 2025 Dec 22;25:1046. doi: 10.1186/s12877-025-06893-7 (PMC12751712; doi:10.1186/s12877-025-06893-7)
Supplement: Supplementary file 1 — Supplementary material 1. [file 12877_2025_6893_MOESM1_ESM.docx]

**APPENDIX**

Appendix 1

Healthcare professional interview topic guide

Knowledge of TEPs

Experience of TEPs

Memorable cases where TEP was important

Impact of TEPs on daily practice

Views on when and why TEPs are important

Views on TEP decision-making

Views on what influences clinical decision-making generally

Views on shared decision-making

Other comments

Patient interview topic guide

How were you feeling before the consultation and why?

- Events leading up to the admission and review by the medical team
- Hopes/expectations for decision-making conversation with the HCP
- Previous conversations about TEP and expectations for this conversation

How did the conversation go?

- Thoughts on decision-making during the consultation
- Understanding of the consultation
- Reactions to the TEP discussion
- Understanding of the TEP discussion
- Feeling heard during the consultation

How are you feeling now?

- Expectations of next steps
- Concerns for the immediate and more distant future
- Opinions on who is responsible for healthcare decision-making

Appendix 2: Analysis

We have used mostly an integrative strategy when presenting fieldnotes, where excerpts and interpretation are woven together (Emerson et al., 2011). This approach allows us to bring together observations from different occasions into a coherent prose, promotes more thematically-focused presentation of the field data and allows a reflective narrative account (Emerson et al., 2011) which seems consistent with our constructivist epistemology wherein we take personal responsibility for generating understanding. We slightly edited the integrated fieldnotes to improve the flow of the text but did not substantially change any of the content: we have used ellipses to indicate where we have removed some words; we have used brackets with an asterisk where we have subsequently added detail for clarity or to remove identifying details. We have expanded acronyms and defined clinical terms. Field notes are denoted in the main text by being written in italics. In some instances, we have included our interpretation of fieldnotes, but we have also left other passages to the reader’s ‘tacit understanding’ whereby they form their own overall interpretation of the data (Emerson et al., 2011).

Appendix 3: Extended fieldnote excerpts

## **CONTEXT: A PRESSURISED ENVIRONMENT**

As I stood there, resus seemed to come to life. There were patients in almost every bay, multiple thrombolysis calls coming in, the stroke team milling around between bays at one end of the department. An announcement went out over the loudspeaker system, carefully dispassionate: “the current wait is 3 hrs 50 minutes, patients are seen in order of priority..” I surveyed the scene absently, not consciously taking in any particular details but submerging myself in the sea of what looked like growing controlled chaos. I vaguely noticed a young boy come in with what could have been his mother and grandfather. In the next-door cubicle, a young male patient was having a chest drain inserted for a pneumothorax. He cried out; the boy winced. Seeing the department though the boy’s eyes, it was a very scary place indeed.

Extended field note, observation in the ED

## **Theme 1: A cog in the hospital machine**

A gastroenterology registrar was frantically trying to book a CT angio^†^ for a patient with gastrointestinal bleed not stabilised by their ’scope. The medical take team, including the consultant, were all at the computers in resus. The slightly heightened sense of activity was reflected in a lengthy Take list on the screen (…) There were lots of patients to see. The atmosphere was focussed, fast paced, although not intensely so. There was a constant movement, coming and going, background dinging of monitors, doors onto the ambulance bay opening and shutting. (…) An 87F with a nasty pneumonia had been moved to the ward before the PTWR. The consultant read the notes quickly and noted that she already had a DNAR from previously. There were lots more on the list so the consultant considered seeing them first when a MET^††^ call went off and we confirmed it was for this patient so the consultant went up to (the ward) and I joined. She walked through the ward, past the SHO at the desk on the phone to the family, straight to the patient in a bay at the back. There were lots of people – radiographer and his portable x-ray machine, two outreach nurses, medical registrar, SHO, F1, relatives of other patients. The patient herself was crumpled up amidst the outreach team, monitoring equipment, pillows and bedsheets. She consultant stood at the end of the bed, took scope, asked if family had been contacted and went out into the corridor to speak with the waiting registrar. The SHO strode past, “I’ve spoken to the family, all done”. The consultant and registrar discussed the case, deliberating the diagnosis – was this all pneumonia or some fluid overload as well? – then the registrar said, “… and regarding ceilings…CPAP^†††^?” the consultant interrupted (I think she misheard CPAP as CPR), “oh no, she’s already not for CPR”. The SpR clarified, “no, CPAP, for the team overnight”. They agreed that as a ceiling of care. She went back to the patient and spoke to the outreach nurses, asking them to review overnight. She confirmed that this was a ward-based ceiling of care, just CPAP. “The family are coming. We can tell them ward based - don’t need to discuss CPAP with them - but you know, her heart, lung, major organs…” They all nodded, plan confirmed. The consultant checked again that the family had been called, everyone said yes, although we didn’t hear what had been discussed. (…) Back in ED resus, greeted by a stressed registrar. Everything felt more intense, faster, even higher pressure. A patient from yesterday’s take, not moved round for more than 24 hrs because of bed pressures, had gone into sudden respiratory failure. “He’s DNAR but he needs NIV^††††^.”

Extended field note, observation on the acute medical take on a medical ward

^†^a medical imaging modality

^††^Medical Emergency Team call, which signals for a clinical team rapidly to attend sick patients

^†††^ Continuous Positive Airway Pressure, a treatment for respiratory failure. This can sometimes be administered on a hospital ward (rather than needing to be admitted to the intensive care unit)

^††††^ Non-invasive ventilation, a treatment for respiratory failure

## **Theme 2: One of many good decisions**

I spoke to one of the AMU ward clerks. (…)she seemed very interested in the DNAR topic, took her responsibilities in it very seriously, spoke passionately, even emotionally. She said that part of her role is booking the transport. She always checks if the patient is DNAR, “it’s about preserving their dignity if that’s the decision. Hopefully the ambulance crew won’t need it, hopefully if 100 people have one they won’t need it! But it they did…” She doesn’t take the nurses’ word for it (tone mildly disparaging), she checks on the EPR (she showed me where, on the EPR header bar). She then goes to the full form and prints it out, puts it in an envelope with the patient details on the front (“we can have so many going all at once, I wouldn’t want it to get confused”) then hands it to the ambulance crew herself and tells them if the patient is DNAR (she stumbled a little over the DNAR/DNACPR acronym). She tries to be discrete, she doesn’t want the patient to hear, “if they haven’t been told, well, not if they haven’t been told but you know if they don’t have capacity or have dementia, or if the family are there, I wouldn’t want someone talking about me like that”. It’s taken her a while to get used to the idea of a DNAR, “you want them to get better,” but she’s learnt that it’s in the patient’s “best interests” – the nurses on the cystic fibrosis ward she used to work on would reassure her about this, “it’s the right thing… it’s the right thing”. … I remarked that she must have seen a lot with the relatives’ room just across from the reception. She agrees, “you hear the laughter, the anecdotes, the sadness”. Sometimes, there is the family of a patient who is end of life and she hears them say, “they look better today”. She looked troubled, “and you know, sometimes that happens before they get worse. I say to them, (she said this carefully, as if it is exactly the phrase she uses) ‘I’m pleased you could see them happy’. I’d hate to say the wrong thing”.

Extended field note, informal interview with ward clerk on the AMU

## **Theme 3: Gatekeeping the conversation**

The F1 presented his clerking of a (female patient in her 60s*) who had come in short of breath and generally unwell. She was recently admitted following a collapse in (…) outpatients, having been referred for suspected malignancy but missed some appointments. Sitting up in her hospital bed in the cubicle, she looked grey, unwell, breathing a little laboured, but smiled at the consultant and they had a back-and-forth conversation. The consultant didn’t go over the history much but focussed on things like a nicotine patch, explaining and reassuring about the CT scan, empathising about not wanting to be in hospital. Going back to the computers to document, she said to the room at large, “Oh dear”. She half turned to me, “so where would this fit in your… thing?” I laughed, ‘I have no opinion!’ She laughed too. In the impression part of the PTWR, she asked the resident doctor to document ‘malignancy and suspected pulmonary embolism’. In REDCOAT^†^, she said full escalation and didn’t qualify. There were only four more to see. Feeling bold, I asked why she chose full escalation. She said, gathering steam as she spoke, “because she was completely independent, and this has been quite a rapid deterioration. Even though if she arrested this could be a terrible outcome, I think she should be intubated, should be ventilated, and then we can put her through a scanner and talk, with all the information, and make a controlled decision. She could have a PE^††^, but we can treat PEs.” She looked me in the eye, “this might be a slow car crash.” I asked, wishing I didn’t have to, and why did you not talk to her about it? “Because if she should be for full escalation…that’s the assumption…I assume that she assumes…I realise that’s a double assumption.. So I wouldn’t talk to her about it. And if I did start talking to her about it, she’s the sort of person who would run a mile”. She said that she didn’t want to have conversations until we knew the scan results. “So there, that’s the answer you knew I’d give”.

Extended field note, PTWR observation in the ED

^†^An audit tool designed to prompt PTWR tasks including TEP

^††^Pulmonary embolism

## **Theme 4: A reluctant conversation**

I spoke with a resident doctor on the ward. She asked me, a little reticently, about who is allowed to do the DNACPR. “I’m sure as F1s we could do it. Discuss, of course, always discuss, but who can put it in? Because last year –” She described a case where she’d felt a patient should be DNAR. “I’d discussed it with family. They weren’t completely happy, but, you know, I said it was a medical decision. But then I spoke to the reg and they said I couldn’t put it on.” I asked why, she looked confused, bothered, “I don’t know, they were just really busy… But then he arrested overnight, and they resuscitated!” The patient hadn’t survived. “The night reg took me on one side in the morning, like. ‘I need to speak to you’. He said, ‘just so you know, that patient had a terrible resuscitation overnight’. I felt like he was saying he should have been DNAR. I was like, I know! He wasn’t being nasty or anything. But just.. I mean, I didn’t think he was going to die overnight or I would have pushed harder.” She seemed troubled, as if she still hadn’t made sense of it. I tried to say the right thing, that sounds really difficult. She couldn’t accept my sympathy, spoke resolutely, “It happens.” She took a breath, “anyway, I guess the take home is…” she paused, then spoke more quickly, tone more rebellious. “Do the DNAR? But I didn’t think he was going to die overnight. I don’t know what the take home would be!” She asked me what I thought about the ‘medical decision’ line when speaking to families. She felt it can worsen conflict (although, I noted, she’d used the phrase earlier). We talked about our approaches to the conversation. Smiling for the first time, she conceded, “I do like a good TEP conversation”.

Extended field note, informal interview with a resident doctor on the AMU

## **Theme 5: Unconstructive conversations**

The next patient was a (female patient in her 70s*) (…) who came in following a fall with a coccyx fracture. They talked about her pain, constipation, she mentioned she doesn’t leave the house nowadays. I didn’t see the TEP conversation coming. He used the same phrasing as with other conversations on the PTWR so far, “when people come into hospital we need to think about if things get worse.. err.. it’s called treatment escalation.. I don’t think that will happen”. The patient looked at him calmly, slightly reluctantly, “resuscitation?” “Yes, has anyone talked to you about that?” No. He didn’t ask how she knew the term. He continued, “I don’t think it would be the right thing for you”. She let him speak, didn’t volunteer much (…) didn’t argue.

I asked him (…) why he made the decision. He said it is her functional status. “I thought she had carers six times a week but she said one, I didn’t know about the leaky valves but that adds to it. Honestly, functional.” He paused, “We probably could get ROSC^†^ but the outcomes – you know, she’s cachectic, bag of bones, I just don’t think..” I asked whether he ever brings patients in on the decision. He said “you can do, but most patients are just not well informed enough. And, you know, I’m well aware that my threshold is –“ he recalled a case where he felt the patient should be DNAR but the ICU team disagreed. “And you could argue that I haven’t done ICU, I don’t have experience of treating anyone with these things. But I’m more qualified than 99.9% of the population”.

I went back to see her (…). She was trying to rest, anxious about her bowels after the enema, thin, very pleasant. My usual opening gambit that she must have seen lots of doctors – oh yes, she’s had hip replacements, knee replacements…. “I don’t say much, I just let them talk. I sometimes don’t know what to say. I’m not the brightest button”. She thought the consultant was good. I asked about the resus discussion. “That was the only bit I wasn’t sure about. I don’t think I’ll sign the form, not until I’ve talked to my son”. She said her husband was in hospital, “during covid, and it was bad with that (the DNAR decisions) then”. He wouldn’t sign the form and that’s how she knows about resuscitation. I asked if she knows what it involves. She said, sadly, “they don’t defibrillate, they just let you go”. She said she doesn’t want to prolong things, she wants to avoid being in hospital. She’s been offered a cardiac bypass and said no, they thought she had gastric cancer a while back and she has a form at home to say she doesn’t want cancer treatment. Her husband was in hospital for a year, came out for 8 months then died of lung cancer. She reiterated that she wants to talk to her son before signing, otherwise he’ll ask why. “I’m all he’s got left since my husband died, well, he has a wife and a new baby. He worries about me, calls me all the time, comes round if I don’t answer”. Since her husband died she has lost confidence and doesn’t go out. I mentioned day centres, they do transport too. “Yes, they do transport, but the thing is I can’t get dressed, I like to have my hair done if I’m going out. Right on cue, the frailty physio arrived.

Observation and informal interviews, PTWR in the ED

^†^Return Of Spontaneous Circulation following a cardiac arrest

Appendix 4: Additional excerpts and extended quotations

| **CONTEXT** | **Illustrative excerpts** |
| --- | --- |
| **A pressurised environment** | The team crowded into one patient’s cubicle, curtains flaying. Kneeling on the floor, the consultant first talked about the diagnosis and plan. Then he said he wanted to have a conversation. The patient seemed confused, he couldn’t hear, said he didn’t have his hearing aids with him. In the monitored bay, alarms were going. There was no emergency and it didn’t feel particularly chaotic. Still, I realised that it was very noisy on the ward, despite the curtains being drawn round. There were still a number of patients waiting to be seen on the round. *Observation, PTWR in the AMU*  It was coming to the end of a busy day on take. The AMU registrar came round to the ED, red faced, breathless, to find the consultant. “The ICU consultant wants to talk to you about B2”. The consultant hadn’t met the patient. He appeared willing but mildly flustered – this didn’t fit with his plan for the final 30 minutes of the PTWR. We saw one more then went round to AMU, walking quickly, to join the registrar back at her computer. She said she didn’t know the patient, pulled up the EPR notes. We looked at the ICU registrar’s documentation – acute kidney injury, acute liver injury, fluid overload. There was a note from her discussion with the ICU consultant: needs consultant to consultant discussions and “careful” decisions re ceiling of care. The medical consultant was anxious to get back to the take team, get them home on time. “Can it wait until after handover?” “Yes, he’s stable”. As we sped back along the corridor, I commented that it’s difficult when you don’t know the patient. He recounted an anecdote of following a colleague’s instructions blindly and a patient dying. “True story. You know, you want to be helpful, but..” *Observation, PTWR in the ED* |
| **THEME** |  |
| **A cog in the hospital machine** | One consultant commented sternly, “not discussing (is)… a disservice to patients”. *Brief interview, medical consultant*  The Trust elearning opened with a slide about the benefits of a CPR decision. It later emphasised the need to talk about dying and facilitating a good death, extending the conversation beyond CPR. The benefits of a DNACPR discussion were listed as follows: ‘Prevents futile and inappropriate attempts at resuscitation in those who are dying; Helps patients and families to understand the seriousness of their condition and to prepare for what lies ahead when broached at appropriate time within patient pathway; Helps to ensure appropriate use of resources; Ensures that decisions are made in a measured way and not in times of crisis; Allows a peaceful and dignified death first two arguments for CPR decisions.’ *Documentary review, Trust eLearning*  The SHO volunteered, nodding vigorously, “yes, I had an awful weekend because no one discussed it.” She said that the lady had an acute kidney injury and heart failure, and then her liver started to fail too. “And it was annoying because it said (on admission*) “would consider single organ support” but she’d been drowsy so they hadn’t talked to her and then “so it was, like, ‘well what if it’s multiorgan support?’ and no one made a decision, and she was so unwell by this point she couldn’t discuss.” She spoke rapidly, the clinical details tumbling after one another, the difficult case clearly still fresh in her mind. *Brief interview, resident doctor* |
| **One of many good decisions** | The night registrar mentioned that there had been one sick one but he’s now in ICU. He seemed to think that was a good thing, hard work done, nothing more for the team to worry about. The consultant thought differently and dressed him down publicly for not calling her, especially as the patient went at 6am so not in the middle of the night. She said it is Trust policy to call the consultant about anyone going to ICU, otherwise if there is a complaint and they don’t know about the case it is not good. It is what we are paid for, she said. She was stern, unrelenting. He was outwardly calm, chipper, soothingly recapping the clinical details and didn’t respond to the critique. Everyone else looked down. *Observation, AMU medical handover*  The resident doctor had anticipated that a DNACPR would be made and had attempted to broach this with a patient’s relative, who resisted. When she told the consultant about this, they looked back through the notes and found that she had a Lasting Power of Attorney. “So the wife has LPA and she wants CPR”. Consultant dictated the following to the resident doctor, who typed: ‘recently for full escalation. LPA wants resus. In view of frailty I believe multiorgan support would not be in his best interests. CPR would need to be reviewed with LPA and a second consultant opinion would be helpful.’ “Let’s hope it’s not me again tomorrow.” *Observation, PTWR in the ED*  The consultant and SHO were reviewing the notes of a (female patient in her 70s*) who had come in confused. The consultant was trying to work out why she was confused, “she doesn’t have any infective symptoms, she’s got Parkinson’s, we’re gonna have to do a PR. She’s dialysis but she passes urine, she could have pyuria, she’ll need an in and out catheter for a sample”.  I walked into resus past a cachectic, grey, frail-looking man on a monitor wearing a facemask and paused, instinctively, to check he was still breathing. I introduced myself to the take registrar. They’d only had one TEP conversation today, and it turned out to be with the man I had just spotted. *Observation, ED*  They suggested that the consultant might want to try (the resus conversation*) again. The consultant was trying to clarify the diagnosis. He said that troponin would remain raised for 10 days so they should check that and the BNP. A hint of reproof that it hadn’t already been checked, did they not know the levels remain raised? He said, “leave him for full escalation until we have the bloods back.” The resident doctors muttered together as he walked off, looking mildly annoyed but smiling and accepting; this didn’t seem a new scenario. I later asked the consultant about his decision, what was his thought process. He said he didn’t feel clear enough about the diagnosis. “Of course you can do it on the basis of their baseline but he lives alone… of course it could all be masked by his family who don’t realise… but it’s difficult.” He remarked, “someone once told me, never have a resus conversation in resus, which I know sounds counter intuitive.” *Observation, PTWR in the ED and brief interview, medical consultant*  Arriving on the AMU, I had just missed a DNAR discussion with family. The consultant said, “she had one in the community. They were fine, they just wanted more information. She nodded as she spoke, I found myself nodding too. *Observation ward round in AMU*  I asked about the other conversations. She said that they were all reinstating prior decisions so she just recapped, told them it was a medical decision. Why do you say it’s a medical decision? “So they don’t feel responsible for their life and death”. *Brief interview, resident doctor* |
| **Gatekeeping the decision** | “Get the decision early, or get ICU down, get everyone together, make a decision,” *Brief interview, medical consultant*  She said that the ward data show that they are performing badly on CPR status, and this can affect accreditation (a local aspirational target for staff, designed to push performance and in part, morale). I asked why that matters? She said that if you score badly overall the interpretation is that care might not be good… and you get unwanted scrutiny which gets in the way of what you are doing. *Brief interview, AMU senior nurse*  I asked her if TEP is part of the handover when a patient comes from ED or another ward. At first, she seemed reticent about this – they have the patient notes open on the computer while taking handover and do check on the EPR header and in the PTWR entry, but it is not a formal part of the handover. Would she ask if they were really old and frail but didn’t have a TEP? No.. She then expanded, seeming more energised: but if the patient was more unwell and she was the nurse in charge or the one receiving the patient, she would push more, ask about ceilings or physiological targets (to they know when to escalate for review), or whether the patient needed an ICU before coming round as “they have more staff over there”. In general, though, she said she could hardly insist on a DNACPR before they come round from ED, implying that the pressures and pace are too fast for that; you can’t have those kinds of stipulations. *Brief interview, senior AMU nurse*  During an admission about six months previously, a CPR form had been filled out as not for CPR but for ICU, not discussed with patient but with a plan to discuss with family. We couldn’t tell from the notes whether if this happened and it had not been revisited on the subsequent admissions. The consultant said, “that’s ok, do that”. The SHO, mildly forceful, fingers poised to type, asked, “so what do you want for REDCOAT?” The consultant replied, quickly, “DNAR.” Then, kindly, offhand, “not for ITU. They won’t take an 85 year old. Consider NIV”. She moved off, the ward round over, towards the AMU office where the daily board round would be starting. I stayed with the SHO who seemed a bit put out. “It’s not ideal, is it?” she said. “We can’t talk to her, don’t know her baseline but she doesn’t have carers… I tried to get a decision. The consultant just said – ” “They won’t take an 85 year old” I filled in. She laughed hollowly, “Yeah exactly. We’re ageist. What if she deteriorates? It’s just kicking the can down the road. I’ve been trying, we had one earlier who’s been here a few days, bedbound, definitely should be DNAR, but she just said, ‘let’s see what happens’”. *Observation, PTWR in the AMU and brief interview with resident doctor*  I can’t count how many patients we saw. TEP seemed on the clerking juniors’ and consultant’s radars and had first place on the PTWR proforma, but it was never discussed with patients. All of the patients were ‘FE’. *Observation, PTWR in the ED*  The registrar said that he had been made DNAR and not for intubation and ventilation during a recent admission, but she wondered about NIV. On the form, it said not discussed with patient due to confusion. The consultant said, “NIV… if they’ve got cognitive problems it can be tricky – dementia and delirium.” It sounded like a “no”. The registrar responded, “he’s not that bad”. Consultant, “Is he not? Ok..” A pause. “Bloods?” We saw the patient in resus, attached to his cardiac monitor. He looked at us over his nasal cannulae with bright eyes. The consultant leaned on the bed rail, seemed, to me, warm, friendly. They talked mostly about the urinary incontinence, which the patient found troublesome especially since the furosemide. The consultant was sympathetic, asked what things were usually like with incontinence, where does he live. The patient answered coherently, did not seem confused. Seamlessly, the consultant continued, “the other thing, whenever anyone comes into hospital, in their best interests, we need to be clear about what we can give and what won’t work. I think things like a breathing machine are probably too much for you to withstand”. The patient said that his breathing had been concerning, although it was better since the diuretics. The consultant continued, “we have treatment for your breathing but I think those things might be too much for you”. The patient seemed in agreement, “I’m 89!” Consultant, “yes, they’re too much for you”. Patient, “I’ll be 90”. Consultant, “Yes, so it’s important to document clearly. The other thing is resuscitation, which is a treatment which might be too much for you. I think this was discussed with your wife when you were last in”. The patient did not seem concerned by this, but clarified, “when was this, the last time?”. A one-line summary of the plan and the team started to leave the bay. The consultant said to the registrar, “Ok, fair enough”. The F1 documented in the REDCOAT DNAR but for NIV, the consultant summarised, “and I think we could do inotropes too. So not for ventilation, not for dialysis, for discussion with ICU”. *Observation, PTWR in the ED (patient male, 80+, moderate/severe frailty, White)*  “you’ve seen me do this very regularly but sometimes, when you keep discussing it with them..” I agreed it’s difficult, “it’s not nice for you or them,” I offered. “It doesn’t matter about me”. He said that he uses the ‘psychological distress’ argument sometimes not to discuss. “They come in to be treated and we say, again and again, ‘we’re not going to resuscitate’, it’s..” He shrugged, rolled his eyes. *Brief interview, medical consultant* |
| **A reluctant conversation** | “They keep asking ‘why, why?”. *Brief interview,* *Senior AMU nurse*  the atmosphere was tense, defensive. The consultant recapped details of the history. The daughter volunteered, “don’t be deceived by her age” and emphasised how well she is. Once they’d talked about the plan, the consultant embarked, “I know my colleague has already spoken to you about resuscitation”. It seemed like the elephant in the room, and the daughter responded, “yes, so I don’t know why it’s being talked about a second time”. The consultant kept an even, pleasant tone. She said it’s good to talk about it. Important to say it doesn’t mean we don’t do other treatment and “just so you know historically it was for people who had a blockage in the heart until it could be unblocked”. The daughter asked if it’s the norm to discuss, “she asked about her age then straightaway about CPR”. *Observation, PTWR in the ED (patient female, 80+ years, mild frailty, White)*  She spent some time with him, explaining what had happened and linking the symptoms with the diagnosis. She asked directly whether he didn’t like doctors (as a way to explain his delayed presentation with a gastrointestinal bleed*) and he said no he just didn’t realise, they both chuckled. He seemed to like her and she was nice with him. She established that he still works, making trophies, and admitted wryly that she’d never heard of them. He exclaimed, “there was I thinking I’m all important!” There was a pause and I thought she was going to conclude but she embarked, one more thing, I need to talk to you and you might find it upsetting so I just want to warn you, it’s about resuscitation. The whole tone changed; her voice more serious. *Observation, PTWR in AMU (patient male, 80+ years, no/mild frailty, White)*  Coming out of a covid isolation room, a consultant tried to recall the conversation for the documentation. “I said, you know, natural course, dignity.” The SHO asked, “hypoxic brain damage?”. “Yes, complications of CPR”. *Observation, PTWR in the ED*  He (the consultant*) recommended stopping the aspirin. She was clear, firm, interjected “Yes, I agree with stopping the aspirin”; it sounded like a formal statement. Pushing on to complete the explanation, he mentioned the risk of stroke. She was brisk, “if it’s going to happen, no one can do anything about that.” *Observation, PTWR in the ED*  She said she had “failed” her last TEP of the night: she talked to him about CPR and he said he wanted it! *Brief interview, resident doctor*  The consultant asked the resident doctor to call the patient’s son to get an impression of what has been discussed before, “if it has, that would be good”. The SHO wanted to know, “with a view to…?” setting a ceiling or not. The consultant said just to understand, for now. I asked the resident doctor about this afterwards, she said, “yeah, I’d rather know because I know that’s what the next question will be. But at least we know, she’s for full escalation." *Observation, PTWR in the ED*  “you feel a oneness with them, you feel they understand your predicament.” One of the ED doctors had it, he was “very impressed”, while a GP visiting his residential home, although not to see him, didn’t. “I’m not saying anything about his credentials. I wouldn’t know, but..” *Brief interview, patient (male, 80+ years, moderate/severe frailty, White)*  I asked her about nurses’ role in TEPs. She looked unsure, I prompted, do they get involved in decisions, conversations, aftermath? She, like others, laughed, “aftermath, yeah”. They don’t get involved in the decisions, “nurses, we know.. but we don’t know all about the scans and things. We can see them, but we don’t know the threshold, what it means.. We just want to support - the patients and the families.” *Brief interview, senior AMU nurse*  She has overheard some of these through the curtains (the old joke about them not being soundproof). She said some are “seasoned” so they know what to say, but others are too “harsh”. She felt it is the communication which upsets people – they know they are old and have health problems but the delivery could be “softer”. I asked how they should say it, she repeated “just more soft”. *Brief interview, student nurse* |
| **Unconstructive conversations** | He thought that if a patient has capacity, they should make the decision and not the family. What about the clinical team? He still thought it should be the patient. “We are a democratic– well, I think we are (he laughed) – 21st century country. You know if it were me, I would want to make the decision. Otherwise how is it ethical?” *Brief interview, ED staff nurse*  I asked her how much she thinks we ‘sell’ the conversation, based on our own beliefs. She looked surprised, hadn’t thought about it, but said yes probably we do, “like, if they’re frail”. We talked about the purpose of involving patients. She said it’s a medical decision, but we want to involve them. I pressed her on this, saying guidelines prompt participation in the decision – she didn’t appear to recognise the guidelines but could appreciate the mismatch. She offered, if you need to be efficient on the ward round it might help to have a plan in mind.. but then laughed, looked comically anguished, “but then we should involve”. *Brief interview, critical care outreach nurse*  They had experience of CPR conversations because the patient’s husband, brother and daughter had been DNAR, so the concept was familiar, she’d made the decision for her husband. I asked about him – he’d had lymphoma then leukaemia, he didn’t like hospitals, she made the DNAR decision because she knew he didn’t want to be in hospital. They all chipped in details of this story and the other DNARs, it seemed an important part of their family narrative. She said she is interested in medical things, “although I’m not a doctor!” like magazines and the television. They make CPR look ok. She doesn’t think her friends would have thought about it, but it doesn’t upset her. I asked her what she knew about what happens during CPR and its aftermath, she said, “not much really.” *Brief interview, patient and family (patient female, 80+ years, mild frailty, White)*  The consultant embarked, “when people come into hospital we need to think about if things get worse.. err.. it’s called treatment escalation.. I don’t think that will happen”. The patient looked at him calmly, slightly reluctantly, “resuscitation?” “Yes, has anyone talked to you about that?” No. He didn’t ask how she knew the term. He continued, “I don’t think it would be the right thing for you”. She let him speak, didn’t volunteer much apart from that she has two leaky heart valves, one beginning with A and one with T (“that’s your aortic and your tricuspid valves”, the consultant explained), didn’t argue. *Observation, PTWR in the ED (patient female, 65-79 years, moderate/severe frailty, White)*  One of the medical consultants was seeing a (female patient in her 90s*) who had come in confused and less mobile. She had decided not to talk to the patient about TEP, felt she was too anxious, but said, “she has capacity, we WOULD discuss escalation, we SHOULD discuss escalation”…The plan nonetheless stated ‘discuss TEP when patient not confused’. The REDCOAT documentation said, ‘Full escalation for now, discussion with patient at present would cause distress. To discuss with NOK to see if any prior discussion with GP regarding DNAR. Can consider NIV and inotropes but multiorgan support and intubation in context of her BMI and frailty would not be in her best interests.’ *Observation, PTWR in the ED*  I spoke with a (male patient in his 90s*) (and his daughter) with weight loss and a likely infection after the PTWR. I asked what makes a good doctor. “Care” said the patient, “like Dr X”. His GP, his daughter explained. “He also likes it when they listen to him”. She’s found that doctors often talk across him to her, but he knows exactly what is going on. He reiterated the names of the GPs he has liked. I asked about the DNAR conversation. The patient didn’t volunteer much about it, just seemed vaguely in agreement. The daughter said she finds it upsetting, but “it’s all about what he wants, if he said jump up and down on his chest I’d be a bit like ‘err!’ (laughing nervously) but that’s what we’d have to do”. I felt I still hadn’t got to the nub of the patient involvement in decision making aspect, but it felt awkward with this pleasant, thin man lying in bed, echoes of what a strong man he had been - ‘an ox’, his daughter said - still a hint of charisma, his daughter so recently in tears. How much choice do you think you have, with the decision, I asked. He smiled, “what will be will be”. His daughter said that’s part of his all faiths and none approach. “Que sera, sera?” I asked. He smiled more broadly, recognising the reference, of his time, “yes”. So how does she feel that the doctors have the conversation? She answered warmly, enthusiastically, “It’s good, I’m pleased someone is thinking about his life”. *Brief interview, patient and NoK (patient male, 80+ years, moderate/severe frailty, Black)*  After speaking about the plan for therapies review, a short admission, she paused for just a second, “Mr X, I want to ask you something. Sorry if it’s a bit unpleasant. Has anyone talked to you about resuscitation?” He answered mildly, “Err.. I think a few doctors have brought it up.. is it like euthanasia?” A nurse appeared at the door with an obs machine and I hurriedly asked her to come back later. The consultant answered firmly, “actually, it’s not euthanasia. If your heart and lungs stop working – essentially you are in the process of dying – we (she mimed) put pressure on the heart.” She said that the history of CPR was if someone had a heart attack but that’s different. She spoke rapidly, I couldn’t catch it all, described some consequences of CPR. I couldn’t work out which way she was selling it, they looked a bit startled, didn’t seem to follow fully, but appeared to be agreeing with whatever it was. “You have a think, talk between you.” The PTWR documentation was for full escalation. When I spoke to the patient and his wife, she told me about his dementia and how difficult it was. I asked him, had he had these conversations before, he’d mentioned euthanasia. He said yes, he didn’t remember how that conversation ended, it wasn’t one of those with all the people in the room. He didn’t seem to recall the CPR conversation with the consultant a few minutes before, although he seemed to recognise my description of a ‘dark haired lady’. I had a dawning realisation from these answers that he probably didn’t have capacity to discuss TEP, despite the PTWR assumption. He volunteered, unexpectedly, “they’ve spent most of their lives doing this”. I asked them both what they’d taken from the conversation, what they thought would happen next. Fractionally less pleasant, the wife replied, “like I said to you, doctor, we’ll talk to the family. Let’s just mention it and see. I don’t want (her husband) worrying about it, and I don’t want me worrying about it”. *Observation, PTWR in the ED (patient male, 80+ years, no/mild frailty, White)*  “I don’t say much, I just let them talk. I sometimes don’t know what to say. I’m not the brightest button”. She thought the consultant was good. I asked about the resus discussion. “That was the only bit I wasn’t sure about. I don’t think I’ll sign the form, not until I’ve talked to my son”. She said her husband was in hospital, “during covid, and it was bad with that (the DNAR decisions) then”. He wouldn’t sign the form and that’s how she knows about resuscitation. I asked if she knows what it involves. She said, sadly, “they don’t defibrillate, they just let you go”. She said she doesn’t want to prolong things, she wants to avoid being in hospital. She’s been offered a cardiac bypass and said no, they thought she had gastric cancer a while back and she has a form at home to say she doesn’t want cancer treatment. *Brief interview, patient (patient female, 65-79 years, moderate/severe frailty, White)*  A (male patient in his 80s*) with fluid on his lungs had seemed in cheerful agreement when told that resuscitation ‘might be too much for (him*)’, remarking, ‘I’m 89!’. When I went back to speak with him, he didn’t recall the PTWR or the consultant, “that wasn’t me!” I prompted that they had discussed, “a plan in case you get more unwell”. He said it had been described not quite like that, but as things we would do, things we wouldn’t do. “Although I can’t remember what they are now!” he reflected, laughing. I said it was going on a ventilator and resuscitation. “Oh yes!” He mimed being bounced up and down by resuscitation. He shook his head comically, I took this as a cue that of course he wouldn’t want them, following his earlier response about his age, said, “and you wouldn’t want those?” He replied, “well, it was more that they wouldn’t be necessary”. I asked what he knew about the treatments, He said, “well, I do know, I have seen, by the time you’re 89 you’ve seen quite a lot” but didn’t elaborate. *Brief interview, patient (male, 80+ years, moderate/severe frailty, White)*  The consultant had kneeled down next to the very fit-looking (female patient in her 80s*) and said that there is something we discuss with everyone, although she looks a very well 87 year old, and that is resuscitation, what does she think about resuscitation? She blustered, was not sure it was ‘appropriate’ to discuss now, he said smoothly that they were ‘happy to offer resuscitation’ and so the documentation was made, for full escalation. I loitered behind to ask permission to talk with her about her experience of conversations with doctors, “Well I don’t like him!” I ended up spending a bit of time around this lady, waiting for her anxious conversation about logistics with her son to conclude, advising on medications, recruiting a nurse to give her analgesia, chatting about her book club and the mayoral election, waiting for observations to be done, finally achieving a signed consent form then being firmly evicted from the room by the porter who had come to take her round to the ward. I tactfully tried to brush past the frailty question on the consent form as irrelevant to her but she interjected, “at 87, I should think I am frail”. Explaining about the finances she needs to sort out and what a disruption it is being in hospital, she commented that she hardly thinks she’s going to live for another seven years. She said this cheerfully but as if she meant it. When I finally tracked her down on the medical ward she looked much older and somehow more crumpled. The curtains were drawn round the bed casting an underwater light. She said it’s not helpful when people just keep repeating the same information. The AMU consultant had, she felt, “a sort of arrogance”. I asked about her ideas on resuscitation. She said that she had been prompted by her daughter to sign one already at home, but when the consultant broached it she thought it inappropriate and “just wanted him to go away”. She signed the DNAR because she doesn’t want a protracted period of dependence. A friend had a stroke but is rich so can afford carers, but she would have to go into a home and “you hear awful things about homes”. She thought it inappropriate to discuss resuscitation when you have had a fracture and expect to recover; it would be appropriate after a stroke if you don’t want to continue a disabled life. I attempted, “of course resuscitation happens after death in both cases…” but she didn’t seem to follow. *Observation, PTWR in the ED and brief interview with patient (patient female, 80+ years, no-/mild frailty, White)*  When the consultant, SHO and I bundled in to see the patient, she said, “I’m deaf”. The consultant checked whether she had a better ear – no – then promised to speak loudly and knelt on the floor with a humorous ‘oomph’. The conversation involved recapping details of the history, symptoms. … A nurse came in, smiling, to give medication in a little white cardboard cup then left. He had stood up by this time to examine her. The consultation seemed to be coming to a close, when he leaned in with one hand against the head of the bed, “since you’ve been in hospital all these times, have you thought about if you become really unwell and your heart stops beating, have you thought about resuscitation?” A priority call announcement boomed over the loudspeaker, drowning his voice, but he persisted. There was a pause, then she said, robustly but defensively, “well I hadn’t, but then for the last surgery someone came and told me, ‘I have to talk to you about this, you know you might not come through this surgery, you can cancel you know’ and I did cancel it, but then the surgeon came and talked to me and I decided to have it, I was feeling so unwell anyway.” She paused, looked a little uncertain. “I’ve got so many things I need to sort out.” The consultant interjected, smiling, reassuring, “I can tell you the medical opinion, we would resuscitate you if your heart stopped”. She didn’t react much, but seemed in agreement. I went back to see her and asked what she liked when doctors spoke to her. She said she doesn’t like “waffle”. She had been surprised when the consultant talked about if her heart stopped. She knows they mentioned it before but this time she thought she would be ok.. I reassured that it is very routine. “I don’t mind him asking, no it’s good they ask, he was very nice”. But she repeated several times as we spoke, “why did he ask?” She mused, “I didn’t think I’d live a long life. You know, I am 73, it’s a good age”. She doesn’t actually want to be “revived”. I asked why not. “I haven’t felt well for the last 10 years”. But, “I’ve got my messy life to sort out, and my dog, I can’t bear to think of him there on his own.” She came back round to the conversation, do they think she is that unwell? She did not seem distressed or agitated, but was not quite able to let it go. “Anyway, there’s more people up there than down here for me now”. She is not religious but feels close to the spirit world. “I had a spirit who used to visit me every night where I used to live. Not an apparition, you know, she used to rattle the bed”. She found it reassuring, like a familiar friend. Her mum comes to her as a spirit too. “I wouldn’t mind going to a different world”. …. I asked what she knew about CPR. She said that when they drop down in the street they do ‘cardiac massage’. She had “a bit of medical training” – she was a dental nurse. But she’s not one of those who wants to keep on living. She had two friends with cancer, one of them in particular just “didn’t want to die”, she’s not like that. I asked if she knows what it’s like after CPR. She initially didn’t understand the question, then, “I don’t know, I’ve never thought about it”. As I thanked her and left, she said, the most energetic she’d been, “you just make sure they do CPR on me!” *Observation, PTWR in the ED and brief interview with patient (patient female, 65-74 years+ years, no-/mild frailty, White)*  There was a (female patient in her 70s*) with severe kidney injury, ‘independent?’ the consultant had confirmed during the notes review. Most of the PTWR consultation was about the plan for investigations. I didn’t foresee a TEP conversation. We made the shuffle to leave and I had actually exited the cubicle, but the consultant spun back and said, abruptly, “This is a conversation we have with everyone.” Her voice was a bit louder, more strained. “My plan was, if your heart stops, we would restart?” The patient looked startled, blank. “You’re not, you’re not against that?” The patient seemed to gather herself together to answer this important question. “No.” A wide-eyed confirmatory shake of the head. I went back and asked directly about the CPR conversation. She’d been “shocked”, had never thought about it.. for herself. “At my stage of life.. I would.. want..” She said this as if making a statement, as if she hadn’t foreseen the need to give an answer but knew that anyway, now, she had to give it, resolutely. Her husband is 10 years older, she explained, and “he was asked and said he didn’t want it”. I asked if she’d thought about it, heard anything, any idea about what happens and the outcomes. She said she’d never thought about it. But she picked me up on the ‘outcomes’ part – “You asked about outcomes. I would hope I would come back.. ok.. As I am”. The questions turned to me, she wanted to know more about it. She asked about downtime, is there a cut off when the outcomes aren’t good? She said she hadn’t thought about it before, and I sensed a dawning and worried appreciation of the uncertainty and possibility that you might not get the outcome you hope for. *Observation, PTWR in the ED and brief interview with patient (patient female, 65-74 years+ years, no-/mild frailty, White)* |

AMU=acute medical unit; BNP=B-type natriuretic peptide; DNAR/DNACPR=do not attempt cardiopulmonary resuscitation; EPR=electronic patient record; ED=emergency department; GP=general practitioner; ICU=intensive care unit; LPA=lasting power of attorney; NIV=non-invasive ventilation; PR=per rectum examination; PTWR=post take ward round; REDCOAT=mnemonic used as an aide-memoire on the PTWR; SHO=senior house officer
